# Avian Influenza A Virus Pandemic Preparedness and Vaccine Development

**DOI:** 10.3390/vaccines6030046

**Published:** 2018-07-25

**Authors:** Rory D. de Vries, Sander Herfst, Mathilde Richard

**Affiliations:** Department of Viroscience, Erasmus MC, P.O. Box 2040, 3000CA Rotterdam, The Netherlands; r.d.devries@erasmusmc.nl (R.D.d.V.); m.richard@erasmusmc.nl (M.R.)

**Keywords:** avian influenza A virus, zoonosis, pandemic, vaccination

## Abstract

Influenza A viruses can infect a wide range of hosts, creating opportunities for zoonotic transmission, i.e., transmission from animals to humans, and placing the human population at constant risk of potential pandemics. In the last hundred years, four influenza A virus pandemics have had a devastating effect, especially the 1918 influenza pandemic that took the lives of at least 40 million people. There is a constant risk that currently circulating avian influenza A viruses (e.g., H5N1, H7N9) will cause a new pandemic. Vaccines are the cornerstone in preparing for and combating potential pandemics. Despite exceptional advances in the design and development of (pre-)pandemic vaccines, there are still serious challenges to overcome, mainly caused by intrinsic characteristics of influenza A viruses: Rapid evolution and a broad host range combined with maintenance in animal reservoirs, making it near impossible to predict the nature and source of the next pandemic virus. Here, recent advances in the development of vaccination strategies to prepare against a pandemic virus coming from the avian reservoir will be discussed. Furthermore, remaining challenges will be addressed, setting the agenda for future research in the development of new vaccination strategies against potentially pandemic influenza A viruses.

## 1. Introduction

Influenza A viruses (IAV) impose a large burden on both human and animal health worldwide. They are exceptional in the diversity of host species that they can infect, including hosts in which circulation is sustained, such as waterfowl (the original reservoir), poultry, humans, swine, horses, dog, and occasional hosts, such as cats, whales, seals, and several other mammalian species [1]. IAV can be categorized into different subtypes based on genetic and antigenic differences in the two surface glycoproteins, the haemagglutinin (HA) and neuraminidase (NA). Wild waterfowl can be infected with viruses harboring combinations of 16 different HA subtypes and nine different NA subtypes. Two novel IAV subtypes (H17N10 and H18N11) have been identified in bats [2]. Inter-species transmission events occur frequently as IAV have a unique capacity to evolve and adapt, gaining the capacity to replicate and spread within a new host species. Direct inter-species transmission of IAV from wild aquatic birds to humans is extremely rare, mainly because of limited contact between humans and wild birds. However, through infection of an intermediate host species to which human exposure is more frequent, such as swine or poultry, transmission to humans becomes more probable. The consequences of the introduction of a novel IAV in an immunologically naive population can be devastating. Upon crossing the animal-to-human transmission barrier, a zoonotic virus must subsequently overcome the human-to-human transmission barrier to eventually become pandemic. In the last hundred years, only four IAV of the A/H1, A/H2 and A/H3 subtypes, gained the ability to be efficiently transmitted between humans. These viruses resulted in the Spanish A/H1N1 pandemic in 1918, the Asian A/H2N2 pandemic in 1957, the Hong Kong A/H3N2 pandemic in 1968, and the A/H1N1 pandemic in 2009 [3,4,5]. Morbidity and mortality associated with IAV pandemics, in combination with economic consequences and the increased emergence of zoonotic avian influenza viruses, emphasize the need for worldwide pandemic preparedness.

## 2. Identifying Pandemic Threats and Enhancing Current Surveillance Programs

A new influenza virus pandemic in the near future is inevitable, although it is uncertain where, when, and from which animal reservoir this pandemic virus will come and of what virus subtype it will be. Initially, avian IAV were thought to have limited ability to directly transmit to humans, and swine were considered a necessary intermediate host species to allow adaptation of avian IAV to mammals. However, direct occasional transmission events of avian A/H5N1 influenza viruses from terrestrial poultry to humans have occurred since 1997, which resulted in a revision of the paradigm that pigs must act as intermediate species for IAV zoonoses [6]. Since the first human cases of avian A/H5N1 virus infection, reports of zoonotic events arising directly from poultry have markedly increased, presumably, in part, as a consequence of increased awareness and surveillance. Human cases of avian IAV infections with the A/H5, A/H6, A/H7, A/H9, and A/H10 subtypes have been reported since [7]. The severity of the disease caused by these zoonotic viruses is variable and is characterized by symptoms ranging from conjunctivitis to influenza-like symptoms, pneumonia associated with acute respiratory distress syndrome, and encephalitis. Among these avian zoonotic influenza viruses, mainly A/H5N1 and A/H7N9 viruses stand out by the number of fatalities they have caused and are of great concern.

Highly pathogenic avian influenza (HPAI) A/H5 viruses from the A/goose/Guangdong/1/1996 (GsGd) lineage emerged in 1997. Since then, they have continued to circulate in poultry in the Eastern hemisphere, causing occasional spillovers to wild birds and mammals, including humans. As of 2 March 2018, 860 laboratory-confirmed human cases of HPAI A/H5N1 virus infection have been reported to the World Health Organization (WHO) since 2003, of which 454 were fatal [8]. Although suspected cases of human-to-human transmission of A/H5 viruses have been described, no sustained transmission between humans has been reported. A/H5 GsGd viruses have diversified into numerous distinct genetic and antigenic clades, and new clades continue to emerge and circulate concurrently. In 2007, an international group of scientists and collaborators, referred to as the WHO/OIE/FAO H5N1 Evolution Working Group, was created to develop a unified nomenclature of H5 GsGd viruses and provide regular updates on their genetic diversification [9]. Moreover, the WHO reports an update on the antigenic characteristics of H5 GsGd viruses that have been circulating for the last six months on a bi-annual basis [10]. However, a global analysis of the antigenic evolution and diversity of A/H5 GsGd has yet to be performed and will be crucial in designing vaccine candidates that induce immunity against antigenically distinct viruses. Moreover, since 2009, reassortant viruses carrying clade 2.3.4.4 HA and internal and NA genes of IAV of different avian origin have been detected, yielding various HA-NA combinations, such as A/H5N1, A/H5N2, A/H5N3, A/H5N5, A/H5N6, and A/H5N8. Of those, A/H5N6 viruses are the only clade 2.3.4.4 viruses that have crossed the species barrier and infected humans. To date, a total of 19 laboratory-confirmed cases of human infection with influenza A/H5N6 virus, including six deaths, have been reported to the WHO since 2014 [11].

Since 2013, A/H7N9 viruses that circulate in poultry in China have caused infections in humans. Until now, six yearly waves of human cases of infection have occurred that resulted in a total of 1625 laboratory-confirmed human infections, including 622 fatalities [12]. The highest number of human cases was recorded during the recent fifth A/H7N9 wave (2017). Notably, some A/H7N9 viruses already harbor mammalian adaption markers that have been associated with increased replication and transmission in mammals or carry amino acid substitutions that contribute to antiviral resistance [13]. However, like A/H5N1 viruses, no sustained transmission between humans has been reported yet. Interestingly, since the beginning of 2017, HPAI A/H7N9 have been detected in poultry and environmental samples but was also isolated from 32 human cases [14]. Moreover, similar to antigenic divergence in A/H5N1 viruses, there is evidence that LPAI A/H7N9 viruses from the fourth wave that belong to the Yangtze River Delta (YRD) lineage, as well as recent HPAI A/H7N9 viruses, have diverged antigenically compared to the early A/H7N9 viruses [15,16].

The possibility that A/H5N1 or A/H7N9 viruses further adapt to humans and subsequently acquire human-to-human transmissibility, either via reassortment with contemporary human viruses or by mutation, underlines the pandemic potential of such zoonotic viruses. These genetic modifications could subsequently enable viruses to cross the final barrier—human-to-human transmission via the airborne route—to become pandemic. In recent years, multiple studies have been conducted to unravel the properties that are required for efficient airborne transmission of zoonotic IAV among humans [17,18,19]. The current state of knowledge predominantly comes from studies on HPAI A/H5N1 viruses, and suggests four minimal requirements for (efficient) airborne transmission of IAV between mammals: (i) efficient attachment of the viral HA glycoprotein to human-type α2,6-linked sialic acid receptors in the upper respiratory tract (rather than the avian-type α2,3-linked sialic acid receptors in the lower respiratory tract); (ii) the loss of a potential N-glycosylation site in HA; (iii) optimal stability of HA, and (iv) increased viral replication through mammalian-adaptation substitutions in the polymerase complex. Although these biological traits favor airborne transmissibility of the tested A/H5N1 viruses, it remains to be elucidated whether these findings can be extrapolated to other A/H5N1 virus clades or even to other virus subtypes such as A/H7N9. However, it is clear that the pandemic threat posed by a zoonotic virus is not solely dictated by its ability to cross the species barrier and achieve efficient transmission between humans. The importance of other factors, such as the level of pre-existing (cross-)immunity in the human population, the length of the infectious period and the likelihood of virus transmission from infected to naive individuals, should not be overlooked should one aim to embrace the complexity of pandemic virus emergence.

Knowledge of the biological properties of airborne-transmissible IAV will allow a better assessment of the public health risk posed by the current, but also future, zoonotic IAV. The inclusion of fairly simple phenotypic assays in ongoing surveillance programs to assess the presence of known airborne transmission traits would improve the screening and identification of circulating pre-pandemic IAV that might require immediate attention and appropriate control actions. Moreover, increasing the capability to rapidly characterize antigenic properties of IAV circulating and emerging in animals will be vital to assess the breadth of protection required from pandemic or universal vaccines.

## 3. Preparing for Future Pandemics

Although antiviral drugs are an important public health countermeasure for treating influenza, vaccination is the most effective public health intervention strategy in the case of a pandemic. The availability of vaccines during a pandemic could potentially abrogate human-to-human transmission and, therefore, prevent the development of new cases. The availability of safe and effective, broadly-acting or universal vaccines will be pivotal in interrupting transmission and preventing severe disease and mortality during a pandemic with a novel IAV. Common vaccination strategies and their respective advantages and disadvantages are described in Table 1.

### 3.1. Challenges to the Development of (Pre) Pandemic Vaccines

A major problem in the development of pre-pandemic vaccines is the difficulty in predicting which zoonotic virus will cause the next pandemic. Although viruses of the A/H5 and A/H7 subtype are under the spotlight, pre-pandemic vaccine development is complicated by the continuous identification of novel zoonotic IAV [1]. Moreover, antigenic evolution and diversity of zoonotic avian IAV pose a serious challenge for vaccine design and pandemic preparedness [9,10,15,16]. To cope with the antigenic diversity of A/H5 GsGd viruses, the WHO has identified 38 vaccine candidates, of which 32 are already available for distribution [10]. For A/H7N9 viruses, three new candidate vaccines have been identified in 2017 to match the antigenic variation observed in recent A/H7N9 viruses [20]. Although this approach is necessary at this stage, the continuous update of candidate virus vaccines is not a sustainable approach for pandemic preparedness against zoonotic avian influenza viruses that might emerge in the near future.

Since it is not possible to predict the nature of a potential future pandemic strain, the gold standard in pandemic vaccine design remains manufacturing an inactivated vaccine that antigenically matches the pandemic influenza virus (Table 1) [21]. Such inactivated vaccines predominantly aim at the induction of virus-neutralizing antibodies directed against HA. However, manufacturing an inactivated vaccine usually takes up to six months (after detection of the pandemic virus), during which the population is vulnerable to infection [22]. This was a lesson learned during the A/H1N1 pandemic of 2009; the preparation of sufficient doses of inactivated vaccine took too long, leading to vaccine availability after the peak of the pandemic in many countries [23]. In addition to the extended time period required for the production of pandemic vaccines, conventional inactivated vaccine formulations against zoonotic avian viruses generally suffer from low immunogenicity compared to seasonal influenza vaccines [24,25,26,27,28,29]. This is partially because these vaccines need to induce a primary immune response in an otherwise immunologically naive population, but also due to the intrinsic low immunogenicity of avian influenza viruses, for reasons that are yet not really understood. To overcome the problem of low immunogenicity of traditional inactivated vaccines, repeated vaccinations and increased antigen doses are often required [30], which do not allow for “dose-sparing” and will increase the chances of vaccine shortages. Adjuvants also need to be used to improve the immunogenicity of vaccines against avian IAV [31].

Collectively, these problems illustrate the need for novel (pre-)pandemic vaccines that can be rapidly produced at a large-scale and confer broad protection against antigenically distinct viruses. The availability of these vaccines will buy time in a pandemic scenario and reduce morbidity and mortality until tailor-made matched vaccines become available. Ideally, these novel vaccines would be truly universal influenza vaccines that offer simultaneous protection against antigenically drifted seasonal viruses, zoonotic influenza viruses, and pandemic influenza viruses [32]. However, initiatives to develop subtype-specific vaccines that offer protection from intrasubtypic drift variants should also be supported as they may be the first step towards universal vaccines. Over the years, several approaches have been taken to develop influenza vaccines capable of eliciting potent, broadly-reactive responses against antigenically diverse strains.

Here, progress made on the front of vaccine development against A/H5 GsGd and A/H7 avian IAV will be discussed. This reflects that current pandemic vaccine development activities are largely focused on viruses of these subtypes. However, ignoring zoonotic IAV of other subtypes would be imprudent, as all zoonotic IAV are presumed to have pandemic potential.

### 3.2. Strategies to Increase the Breadth and the Height of the Antibody Response upon Vaccination

Vaccination with a single (adjuvanted) H5 antigen can elicit cross-clade reactivity, and even in some cases, cross-clade protection in the absence of hemagglutination-inhibition (HI) antibodies [33,34,35,36]. However, it is difficult to predict whether an A/H5 antigen will protect against A/H5 viruses from distinct clades. Vaccination with a mixture of antigens from different H5 GsGd clades has been explored in animal models as a multivalent vaccination approach while utilizing different vaccine platforms: whole inactivated vaccines [37], DNA vaccines [38] or baculovirus vectors [39]. In general, multivalent vaccines induced broader antibody responses than monovalent vaccines. Additionally, heterologous prime-boost strategies using antigenically divergent strains have also been evaluated to broaden the immune response against distinct A/H5 GsGd viruses. Recent studies were performed to assess whether existing pre-pandemic stockpiled A/H5 vaccines could be used in heterologous prime-boost vaccination regimes to provide protection against the newly emerged divergent clade 2.3.4.4 viruses. Priming with AS03-adjuvanted split inactivated A/Vietnam/1203/2004 (clade 1) and boosting with A/Anhui/1/05 (clade 2.3.4) slightly improved the cross-reactivity with clade 2.3.4.4 viruses in ferrets compared to a homologous prime-boost regime, albeit without any statistical significance [40]. Similar heterologous prime-boost vaccination schemes were performed in humans (A/Vietnam/1203/2004 (clade 1) followed by A/Anhui/1/05 (clade 2.3.4)) which led to a modest level of HI antibodies cross-reactive with clade 2.3.4.4 viruses, while homologous prime-boost (A/Vietnam/1203/2004 (clade 1)) did not [41].

Several clinical studies have highlighted the timing between the prime and boost as a critical parameter for inducing optimal immunity. An interval of six months led to higher and broader immune responses when compared to a 28-day interval [42,43]. Unfortunately, a six-month interval between prime and boost is not conceivable in a pandemic context, unless the population had been primed before the onset of the pandemic. Although this is an interesting approach, the benefit of pre-pandemic priming of the population depends on the possibility to induce lasting immunity after vaccination. It has been shown that ferrets can be protected against A/H5 viruses for up to 15 months after vaccination with inactivated vaccines [44,45]. In humans, subjects who were primed with a clade 0 virus at least 6–8 years before the boost vaccination still demonstrated a secondary immune response upon administration of a clade 1 vaccine as compared to unprimed subjects [46,47], suggesting that pre-pandemic priming could be a useful strategy for pandemic control. However, several parameters remain to be investigated to maximize the immune responses induced by heterologous prime-boost vaccination regimens, such as the order of prime and boost in the context of the “original antigenic sin” and “back-boost” phenomena [48]. Furthermore, the antigenic distance between the prime and boost vaccine will be important for inducing optimal immunity. Further understanding of the underlying immune mechanisms of prime-boost will help to design better vaccination strategies utilizing both stockpiled antigens or newly developed vaccines for pandemic preparedness. Although approaches meant to combine the immune responses against antigenically distinct strains are promising for inducing broad responses against viruses of different clades, their limitation lies in the necessity to produce and stockpile multiple antigens, which in a pandemic situation might limit the number of doses available.

Therefore, the field of vaccine development has moved forward to the rational design of antigens that could induce broad antibody responses, for example, ancestral HA and NA proteins or sequences [49], consensus sequences [50,51,52,53,54], and centralized HA proteins [55]. Putative HA and NA ancestral proteins, representing the most recent common ancestor of a selection of viruses, were generated to represent the complete population of circulating A/H5 GsGd viruses. The feasibility of this approach was determined using whole inactivated vaccines in ferrets. However, the ancestral antigens were not superior in terms of protection compared to wild-type antigens [49]. Whereas ancestral sequences are expected to be an estimation of a sequence that once existed in the past, consensus sequences encoded for the most common amino acid found at each position and are expected to be more representative of the currently circulating strains. In a DNA vaccination approach (Table 1), consensus-based A/H5 sequences were shown to elicit broadly-reactive humoral and cellular immune responses [50,51,52]. However, the design of consensus antigens is inherently biased by the nature of the input sequences and by the fact that certain A/H5 clades are overrepresented in the sequence databases. A computationally optimized, broadly-reactive antigen (COBRA) design approach was developed to avoid this sample bias. A COBRA clade 2 A/H5 antigen was designed using multiple rounds of consensus generation [53] and was expressed on virus-like particles as vaccine immunogen (Table 1). Ferrets vaccinated with the COBRA clade 2 virus-like particle developed a broader immune response against viruses of subclades 2.1, 2.2, and 2.3 than those vaccinated with a wild-type clade 2.2 antigens. However, a single A/H5 COBRA virus-like particle vaccine, designed using all sequences from human and avian A/H5N1 influenza viruses available, could not elicit antibody responses against all A/H5 clades in mice [54]. However, vaccination of mice with a cocktail of three COBRA A/H5 virus-like particle vaccines in a prime-boost strategy induced a broadly-reactive set of antibodies that recognized A/H5N1 viruses from 11 clades/subclades isolated over a 12-year span [54]. Webby and colleagues took an approach similar to COBRA, in which the sample bias was corrected by selecting representative sequences of each major branch of the A/H5 phylogenetic tree. Mice vaccinated with A/H5-consensus protein expressed in an adenovirus vector were protected from lethal challenge against several A/H5 viruses from antigenically divergent clades [55].

Although these synthetic antigens have been able to successfully elicit broader antibody responses than antigens derived from a wild-type virus, their design is solely based on genetic information. Synthetic consensus antigens are, therefore, actually representative of a population of sequences and not necessarily of the antigenic properties of a population of viruses. Koel et al. showed that antigenic differences within H5 GsGd clade 2.1 were primarily caused by a few amino acid changes immediately adjacent to the receptor binding site [56] and were not necessarily predictable from (phylo)genetic information alone. A global view of the antigenic diversity and evolution of H5 GsGd viruses combined with the identification of the molecular determinants that are responsible for antigenic diversity within H5 GsGd lineage viruses would contribute to the design of an antigenically central HA that could elicit broad immunity against viruses of different clades.

Glycosylation is an important factor in the folding, function, and antigenicity of HA and glycan-masking is thought to be an important mechanism for IAV to escape recognition by antibodies. The glycosylation state of HA, both in term of quantity and quality, affects immunogenicity and modulates the induction of cross-reactive antibody responses [57,58]. Antibodies raised against an HA protein with only a single N-linked glycan at each glycosylation site had better binding affinity and neutralizing activity than antibodies raised against the fully glycosylated HA protein [59]. Notably, glycosylation has also been used as a tool to redirect antibody responses towards the more conserved HA stem [60,61]. The idea is that by hyperglycosylating the head of HA, variable antigenic sites become shielded, and antibodies directed against antigenic sites in the HA stem would predominantly be induced. Similarly, antigens with an altered glycosylation state have been used to induce antibodies directed against more conserved parts of the HA head [61], like the receptor binding site which has been shown to be the target of cross-reactive antibodies [62]. On the other hand, it has been reported that vaccination with a fully deglycosylated HA decreased HA-specific antibody levels [63] and that glycosylation could enhance the recognition by lectins of the immune system [58]. Altogether, balancing the glycosylation state of the HA used as antigen in vaccines would be useful when developing an HA-based vaccine.

In parallel, changing receptor specificities of vaccine viruses influences the induction of immune responses. Studies have shown that live attenuated influenza vaccine viruses (Table 1) with dual receptor binding specificity were able to elicit better immune responses in ferrets due to increased replication in the upper respiratory tract when compared to vaccine viruses with only α2,3-SA binding specificity [64]. The impact of receptor binding preference on immunogenicity of whole inactivated vaccines has also been investigated [65,66]. Hoffmann et al. showed that introduction of the S223N (H5 numbering starting from the signal peptide [67]) mutation (expected to change the receptor binding preference from avian- to human-like receptors) in a whole inactivated A/H5 vaccine, increased its immunogenicity in ferrets [66]. Similarly, in whole inactivated vaccines of the A/H1 subtype, the change from avian to human-like receptor binding specificity also increased antibody titers in ferrets and mice upon vaccination [65]. There is additional evidence to support that mutations increasing the thermal and pH stability of HA might also increase the immunogenicity of vaccines [68] and also the yield and long-term stability of vaccines [69].

Approaches described above all aim at the induction of hemagglutination inhibition (HI) and virus neutralizing (VN) antibodies targeting the globular head of HA. However, this is the domain that harbors the substitutions that define antigenic drift [70] and HA head-specific antibodies, therefore, often fail to cross-neutralize viruses of alternative subtypes. It is generally thought that a universal vaccine, or even a vaccine targeting intrasubtypic drift variants, should induce antibodies targeting more conserved proteins or regions of the HA glycoprotein. Potential targets could be M2e (the ectodomain of the IAV matrix protein 2, highly conserved in both avian and human IAV) [71], the HAstalk [72] or NA [73,74]. Importantly, it should be kept in mind that antibodies directed against these targets often do not directly neutralize IAV and do not offer sterile immunity. Rather, antibody-dependent cellular cytotoxicity (ADCC) and antibody cellular phagocytosis (ADP) play an important role as a correlate of protection mediated by M2e- and HAstalk-specific antibodies [75,76,77], whereas NA-specific antibodies predominantly prevent viral budding [78]. Therefore, the presence of virus-specific antibodies not mediating direct virus neutralization still allow low-level virus replication, with potential development of clinical signs and transmission.

### 3.3. Activating the Cellular Arm of the Immune Response

In addition to the induction of neutralizing and non-neutralizing antibodies targeting mainly antigens displayed on the surface of the virion, vaccines inducing broadly-reactive responses should also activate the cellular arm of the immune system. It has been shown that IAV infection can induce heterosubtypic immunity, which largely correlated with the induction of cross-reactive T-cells [79]. The majority of cross-reactive T-cells, predominantly CD8^+^ cytotoxic T-cells, is directed against conserved internal proteins, like the nucleoprotein (NP), matrix protein (M1) or polymerase complex proteins (PA, PB1, and PB2) [79]. Since human CD8^+^ T-cells induced by natural seasonal IAV infection naturally cross-react with viruses of the A/H5N1, A/H7N9, and swine-origin pandemic A/H1N1 subtypes [80,81,82], induction of cross-reactive T-cell responses seems appealing as a mode of action for universal influenza vaccines. Indeed, during the 2009 pandemic, subjects with a high level of pre-existing IAV-specific CD8^+^ T-cells were less likely to develop severe disease after infection [83]. Similar to M2e-, HAstalk- or NA-specific antibodies, virus-specific T-cells cannot provide sterile immunity but could significantly contribute to clinical protection against antigenically drifted seasonal viruses, zoonotic influenza viruses, and pandemic influenza viruses.

It has been established that inactivated vaccines inefficiently induce CD8^+^ T-cell responses [84]. For efficient induction of virus-specific CD8^+^ T-cells, vaccine delivery systems that allow de novo synthesis of viral proteins in vivo are required. Several vaccination platforms would be suitable to achieve this, such as live attenuated vaccines, virus-like particles, DNA vaccines or vector-based vaccines (Table 1). To this end, cold-adapted vaccines or pseudotyped replication-deficient influenza vaccines have been developed for A/H5 viruses and evaluated in animal models and phase I clinical trials [85,86]. They elicited strong CD8^+^ T-cells responses and protected mice and ferrets from challenge with homologous and heterologous viruses [87,88]. Evaluation in phase I clinical trials revealed that, although live attenuated vaccines failed to elicit robust antibody responses [87], they primed long-term immune memory that could be boosted upon administration of an inactivated vaccine one to five years later [86,89]. Live attenuated vaccines were successful in inducing T-cell responses directed to internal proteins, as well as HA-specific T-cell responses to seasonal H1 and H3 influenza viruses. These HA-specific T-cells showed limited cross-reactivity with HA from A/H5N1, suggesting an “original antigenic sin” phenomenon [90]. It has been shown that virus-like particles containing HA, NA, and M1 induced significantly higher cell-mediated immune responses in mice than whole-inactivated vaccines [91]. In a phase I clinical trial, DNA vaccines encoding H5 HA, NP and M2 elicited H5-specific T-cells in most subjects [92].

Vector-based influenza vaccines, mainly recombinant adenoviruses and poxviruses, are also promising vaccine candidates (Table 1). Most viral vectors are regarded as “live” vaccines, but are severely replication-restricted or even completely replication-deficient in humans, leading to an excellent safety profile even in the immunocompromized. Additionally, viral vectors can generally be propagated to high viral titers, fulfilling the pressing need for (pre-)pandemic immunogenic vaccines that can be rapidly produced at a large-scale. A well-described candidate vaccine vector is modified vaccinia virus Ankara (MVA), originally developed as a safe and replication-deficient smallpox vaccine [93]. MVA drives endogenous antigen production in infected cells, leading to efficient antigen processing and presentation, and subsequent induction of antigen-specific B- and T-cell responses. Since the generation of recombinant (r)MVA, by insertion of genes encoding antigens of interest into the viral genome is relatively easy, a number or rMVA vaccine candidates expressing influenza A virus NP, M1, M2, PB2, NA, and HA have been successfully tested in mouse, ferret and macaque vaccination-challenge experiments [94,95].

Recombinant MVA vaccines expressing the HA genes from various A/H5N1 influenza viruses have been constructed and tested for protective efficacy against challenge viruses belonging to different antigenic clades in animal models [95]. It has been shown that a rMVA expressing HA from the clade 1 A/H5N1 virus A/Vietnam/1194/04 can offer protection from homologous challenge, in addition to protection from several antigenically distinct A/H5N1 viruses in mice and macaques [96,97,98,99,100]. In mice, the rMVA-H5 vaccine induced high levels of HA-specific CD4 and CD8 T-cells targeting epitopes that are conserved among different HA clades [98]. This successful induction of broadly-reactive T-cells in animal models led to the only reported phase I/IIa clinical trial with an MVA-HA-based (pre-)pandemic vaccine that we know of [101]. Not only was this rMVA expressing A/H5 from A/Vietnam/1194/04 capable of inducing homologous responses, but antibodies were also induced that cross-reacted with heterologous A/H5N1 and newly-emerging A/H5N6 and A/H5N8 viruses [101,102]. Candidate MVA-based (pre-)pandemic vaccines targeting A/H7N9 have only been tested in animal models to a limited extent. Vaccination of ferrets with rMVA expressing A/H7 from A/Shanghai/2/13 offered partial protection from challenge with a closely related A/H7N9 virus [103].

MVA does not only allow for expression of a single wildtype antigen, but multiple antigens can also easily be expressed that can be modified for optimal induction of cross-reactive immune responses. MVA vaccines expressing a combination of HA and NP, or HA and NA, of an A/H5N1 virus were shown to be (partially) protective against homologous and heterologous challenge in mice [104,105]. In an attempt to generate a rMVA affording intrasubtypic protection from various A/H5N1 viruses, Kamlangdee et al. constructed a so-called MVA-H5Mosaic vaccine virus similar to the above described common ancestor and COBRA antigens. This MVA expressed a computationally generated mosaic H5 gene reflecting gene sequences of 2,145 A/H5N1 isolates. Vaccination of mice with this vaccine candidate led to antibodies that cross-reacted with A/H5N1 viruses of all clades, and afforded protection from both A/H5N1 and A/H1N1 challenge infection [106].

## 4. Conclusions

The generation of novel vaccines that are able to overcome the challenges of IAV antigenic diversity and inherent low immunogenicity of vaccines against avian IAV viruses is a crucial step in pandemic preparedness. Despite recent progress in development and design of vaccines against pandemics threats, several points still merit the attention of the influenza virus community. Increased knowledge on the antigenic diversity of influenza viruses that currently circulate in animal reservoirs is vital to assess the breadth of protection required from future vaccines. To assess the breadth of the immune response elicited upon vaccination, human sera obtained from clinical studies should be evaluated against a selected panel of viruses representing the current antigenic diversity of avian IAV.

Animal models to study vaccine efficacy should be further developed and optimized to facilitate the extrapolation of the data from pre-clinical studies to clinical studies. One critical point here is the obvious lack of immunological tools for species other than mice. As ferrets are regarded the “gold standard” as influenza animal model, there is an urgent need to develop antibodies and novel tools to study the immunology of ferrets. Another limitation of currently performed pre-pandemic vaccine studies in animal models is that, to date, there are no models to accurately study the impact of pre-existing immunity against A/H1, A/H2, and A/H3 subtypes on the efficacy of pre-pandemic vaccines. All vaccines are currently evaluated pre-clinically in immunologically naive animal models, limiting the potential for translation to the human population that naturally has influenza virus-specific immunity. The immunological correlates protecting from disease caused by pandemic influenza viruses are less well understood than those that protect from seasonal influenza virus infection. For seasonal influenza viruses, an HI-titer above 40 has been associated with an approximately 50% reduction in infection with seasonal influenza viruses in humans [107] and is widely considered to be seroprotective. However, it remains to be determined what the minimal level of HI antibodies that correlates with protection against pandemic influenza viruses is and even whether such a correlation exists at all. Additionally, especially with the design of novel pre-pandemic vaccines that induce non-neutralizing antibodies or T-cells in mind, research is urgently required to determine whether these correlate to protection from zoonotic IAV at all. This work should start with the standardization of non HI-assays, such as determining levels of antibodies that mediate ADCC, direct virus neutralization assays, and T-cell assays. This will aid in the design of robust clinical trials that will pave the way for the development of regulatory requirements for approval and licensing of novel vaccine platforms. It has become evident over the years that the type of vaccine platform has a great impact on the immunological pathways elicited upon vaccination.

In recent years efforts have been made to design universal vaccines focusing on induction of either a broad B- or a T-cell immune response. In the future, to address the challenge of vaccine design for pandemic preparedness, research should focus on developing new multivalent pre-pandemic vaccines that elicit both broad B- and T-cells responses. This could be achieved by combining vaccine platforms [108]. Another approach would be the combination of rational antigen design meant to induce broad humoral responses with an adequate vaccine platform that elicits cellular responses. Efforts to combine both arms of the immune response will be crucial to develop efficient (pre-)pandemic vaccines against zoonotic IAV.

## Figures and Tables

**Table 1:** Advantages and disadvantages of various vaccination strategies.

Vaccine Platform	Advantages	Disadvantages	References
Inactivated vaccines	Safe	Limited immunogenicity Short-lasting immunity Often multiple doses required Poor induction cellular immunity	[21,22,24,30]
Subunit vaccines	Safe Selection of antigen possible	Limited immunogenicity Short-lasting immunity Poor induction cellular immunity	[27,40,41]
Live attenuated vaccines	Present conformational epitopes Single dose sufficient Induction cellular immunity	Safety risk in immunocompromized Reversion to wildtype possible Interference by maternal antibodies	[26,64,85,86,88]
Virus-like particles	Safe Present conformational epitopes	Complicated production process	[53,54,91]
DNA vaccines	Present conformational epitopes Selection of antigen possible Induction cellular immunity Effective in heterologous prime-boost	Often poorly protective when used exclusively	[38,50,51,52,92]
Vector-based vaccines	Safe Present conformational epitopes Selection of antigen possible Induction humoral and cellular immunity Effective in heterologous prime-boost	Often multiple doses required Interference by vector-specific immunity	[55,94,96,99,101]

## References

[1] Short K.R., Richard M., Verhagen J.H., van Riel D., Schrauwen E.J., van den Brand J.M., Manz B., Bodewes R., Herfst S. (2015). One health, multiple challenges: The inter-species transmission of influenza a virus. One Health.

[2] Tong S., Li Y., Rivailler P., Conrardy C., Castillo D.A., Chen L.M., Recuenco S., Ellison J.A., Davis C.T., York I.A. (2012). A distinct lineage of influenza a virus from bats. Proc. Natl. Acad. Sci. USA.

[3] Taubenberger J.K., Morens D.M. (2006). 1918 influenza: The mother of all pandemics. Emerg. Infect. Dis..

[4] Scholtissek C., Rohde W., Von Hoyningen V., Rott R. (1978). On the origin of the human influenza virus subtypes H2N2 and H3N2. Virology.

[5] Neumann G., Noda T., Kawaoka Y. (2009). Emergence and pandemic potential of swine-origin H1N1 influenza virus. Nature.

[6] Subbarao K., Katz J. (2000). Avian influenza viruses infecting humans. Cell. Mol. Life Sci..

[7] Richard M., de Graaf M., Herfst S. (2014). Avian influenza a viruses: From zoonosis to pandemic. Future Virol..

[8] WHO Cumulative Number of Confirmed Human Cases for Avian Influenza a(H5N1) Reported to WHO, 2003–2018. http://www.who.int/influenza/human_animal_interface/2018_03_02_tableH5N1.pdf?ua=1.

[9] WHO/OIE/FAO H5N1 Evolution Working Group (2008). Toward a unified nomenclature system for highly pathogenic avian influenza virus (H5N1). Emerg. Infect. Dis..

[10] WHO Antigenic and Genetic Characteristics of Zoonotic Influenza Viruses and Candidate Vaccine Viruses Developed for Potential Use in Human Vaccines. http://www.who.int/influenza/vaccines/virus/characteristics_virus_vaccines/en/.

[11] WHO Avian Influenza Weekly Update Number 631. http://www.wpro.who.int/emerging_diseases/ai_weekly_631_wpro_20180406.pdf.

[12] FAO H7N9 Situation Update. http://www.fao.org/ag/againfo/programmes/en/empres/H7N9/situation_update.html.

[13] Quan C., Shi W., Yang Y., Yang Y., Liu X., Xu W., Li H., Li J., Wang Q., Tong Z. (2018). New threats of H7N9 influenza virus: The spread and evolution of highly and low pathogenic variants with high genomic diversity in wave five. J. Virol..

[14] Zhang F., Bi Y., Wang J., Wong G., Shi W., Hu F., Yang Y., Yang L., Deng X., Jiang S. (2017). Human infections with recently-emerging highly pathogenic h7n9 avian influenza virus in china. J. Infect..

[15] WHO Antigenic and Genetic Characteristics of Zoonotic Influenza Viruses and Development of Candidate Vaccine Viruses for Pandemic Preparedness. http://www.who.int/influenza/vaccines/virus/201703_zoonotic_vaccinevirusupdate.pdf?ua=1.

[16] Yang L., Zhu W., Li X., Chen M., Wu J., Yu P., Qi S., Huang Y., Shi W., Dong J. (2017). Genesis and spread of newly emerged highly pathogenic H7N9 avian viruses in mainland china. J. Virol..

[17] Herfst S., Schrauwen E.J., Linster M., Chutinimitkul S., de Wit E., Munster V.J., Sorrell E.M., Bestebroer T.M., Burke D.F., Smith D.J. (2012). Airborne transmission of influenza A/H5N1 virus between ferrets. Science.

[18] Imai M., Watanabe T., Hatta M., Das S.C., Ozawa M., Shinya K., Zhong G., Hanson A., Katsura H., Watanabe S. (2012). Experimental adaptation of an influenza H5 HA confers respiratory droplet transmission to a reassortant H5 HA/H1N1 virus in ferrets. Nature.

[19] Linster M., van Boheemen S., de Graaf M., Schrauwen E.J., Lexmond P., Manz B., Bestebroer T.M., Baumann J., van Riel D., Rimmelzwaan G.F. (2014). Identification, characterization, and natural selection of mutations driving airborne transmission of A/H5N1 virus. Cell.

[20] WHO Summary of Status of Development and Availability of Avian Influenza A(H7N9) Candidate Vaccine Viruses and Potency Testing Reagents. http://www.who.int/influenza/vaccines/virus/candidates_reagents/summary_a_h7n9_cvv_20180305.pdf?ua=1.

[21] WHO Sage Working Group on Influenza Vaccines and Immunizations Influenza A(H5N1) Vaccine Stockpile and Inter-Pandemic Vaccine Use. http://www.who.int/immunization/sage/meetings/2013/november/SAGE_WG_H5vaccine_background_paper_16Oct2013_v4.pdf.

[22] Krammer F., Palese P. (2015). Advances in the development of influenza virus vaccines. Nat. Rev. Drug Discov..

[23] Skowronski D.M., Janjua N.Z., De Serres G., Hottes T.S., Dickinson J.A., Crowcroft N., Kwindt T.L., Tang P., Charest H., Fonseca K. (2011). Effectiveness of AS03 adjuvanted pandemic h1n1 vaccine: Case-control evaluation based on sentinel surveillance system in canada, autumn 2009. BMJ.

[24] Cox R.J., Madhun A.S., Hauge S., Sjursen H., Major D., Kuhne M., Hoschler K., Saville M., Vogel F.R., Barclay W. (2009). A phase I clinical trial of a PER.C6 cell grown influenza H7 virus vaccine. Vaccine.

[25] Mulligan M.J., Bernstein D.I., Winokur P., Rupp R., Anderson E., Rouphael N., Dickey M., Stapleton J.T., Edupuganti S., Spearman P. (2014). Serological responses to an avian influenza A/H7N9 vaccine mixed at the point-of-use with MF59 adjuvant: A randomized clinical trial. JAMA.

[26] Talaat K.R., Karron R.A., Callahan K.A., Luke C.J., DiLorenzo S.C., Chen G.L., Lamirande E.W., Jin H., Coelingh K.L., Murphy B.R. (2009). A live attenuated H7N3 influenza virus vaccine is well tolerated and immunogenic in a phase I trial in healthy adults. Vaccine.

[27] Stephenson I., Nicholson K.G., Gluck R., Mischler R., Newman R.W., Palache A.M., Verlander N.Q., Warburton F., Wood J.M., Zambon M.C. (2003). Safety and antigenicity of whole virus and subunit influenza A/Hong Kong/1073/99 (H9N2) vaccine in healthy adults: Phase I randomised trial. Lancet.

[28] Nicholson K.G., Colegate A.E., Podda A., Stephenson I., Wood J., Ypma E., Zambon M.C. (2001). Safety and antigenicity of non-adjuvanted and MF59-adjuvanted influenza A/Duck/Singapore/97 (H5N3) vaccine: A randomised trial of two potential vaccines against H5N1 influenza. Lancet.

[29] Stephenson I., Bugarini R., Nicholson K.G., Podda A., Wood J.M., Zambon M.C., Katz J.M. (2005). Cross-reactivity to highly pathogenic avian influenza H5N1 viruses after vaccination with nonadjuvanted and MF59-adjuvanted influenza A/Duck/Singapore/97 (H5N3) vaccine: A potential priming strategy. J. Infect. Dis..

[30] Couch R.B., Decker W.K., Utama B., Atmar R.L., Nino D., Feng J.Q., Halpert M.M., Air G.M. (2012). Evaluations for in vitro correlates of immunogenicity of inactivated influenza a H5, H7 and H9 vaccines in humans. PLoS ONE.

[31] Liu F., Sun X., Fairman J., Lewis D.B., Katz J.M., Levine M., Tumpey T.M., Lu X. (2016). A cationic liposome-DNA complexes adjuvant (JVRS-100) enhances the immunogenicity and cross-protective efficacy of pre-pandemic influenza A (H5N1) vaccine in ferrets. Virology.

[32] Berlanda Scorza F., Tsvetnitsky V., Donnelly J.J. (2016). Universal influenza vaccines: Shifting to better vaccines. Vaccine.

[33] Baras B., Stittelaar K.J., Simon J.H., Thoolen R.J., Mossman S.P., Pistoor F.H., van Amerongen G., Wettendorff M.A., Hanon E., Osterhaus A.D. (2008). Cross-Protection against lethal H5N1 challenge in ferrets with an adjuvanted pandemic influenza vaccine. PLoS ONE.

[34] Govorkova E.A., Webby R.J., Humberd J., Seiler J.P., Webster R.G. (2006). Immunization with reverse-genetics-produced H5N1 influenza vaccine protects ferrets against homologous and heterologous challenge. J. Infect. Dis..

[35] Lipatov A.S., Hoffmann E., Salomon R., Yen H.L., Webster R.G. (2006). Cross-protectiveness and immunogenicity of influenza A/Duck/Singapore/3/97(H5) vaccines against infection with A/Vietnam/1203/04(H5N1) virus in ferrets. J. Infect. Dis..

[36] Park S.J., Si Y.J., Kim J., Song M.S., Kim S.M., Kim E.H., Kwon H.I., Kim Y.I., Lee O.J., Shin O.S. (2016). Cross-protective efficacies of highly-pathogenic avian influenza H5N1 vaccines against a recent H5N8 virus. Virology.

[37] Forrest H.L., Khalenkov A.M., Govorkova E.A., Kim J.K., Del Giudice G., Webster R.G. (2009). Single- and multiple-clade influenza A H5n1 vaccines induce cross protection in ferrets. Vaccine.

[38] Rao S., Kong W.P., Wei C.J., Yang Z.Y., Nason M., Styles D., DeTolla L.J., Panda A., Sorrell E.M., Song H. (2008). Multivalent HA DNA vaccination protects against highly pathogenic H5N1 avian influenza infection in chickens and mice. PLoS ONE.

[39] Prabakaran M., He F., Meng T., Madhan S., Yunrui T., Jia Q., Kwang J. (2010). Neutralizing epitopes of influenza virus hemagglutinin: Target for the development of a universal vaccine against H5N1 lineages. J. Virol..

[40] Sun X., Belser J.A., Pulit-Penaloza J.A., Creager H.M., Guo Z., Jefferson S.N., Liu F., York I.A., Stevens J., Maines T.R. (2017). Stockpiled pre-pandemic H5N1 influenza virus vaccines with AS03 adjuvant provide cross-protection from H5N2 clade 2.3.4.4 virus challenge in ferrets. Virology.

[41] Levine M.Z., Holiday C., Liu F., Jefferson S., Gillis E., Bellamy A.R., Tumpey T., Katz J.M. (2017). Cross-reactive antibody responses to novel H5Nx influenza viruses following homologous and heterologous prime-boost vaccination with a prepandemic stockpiled A(H5N1) vaccine in humans. J. Infect. Dis..

[42] Schwarz T.F., Horacek T., Knuf M., Damman H.G., Roman F., Drame M., Gillard P., Jilg W. (2009). Single dose vaccination with AS03-adjuvanted H5N1 vaccines in a randomized trial induces strong and broad immune responsiveness to booster vaccination in adults. Vaccine.

[43] Belshe R.B., Frey S.E., Graham I., Mulligan M.J., Edupuganti S., Jackson L.A., Wald A., Poland G., Jacobson R., Keyserling H.L. (2011). Safety and immunogenicity of influenza A H5 subunit vaccines: Effect of vaccine schedule and antigenic variant. J. Infect. Dis..

[44] Ducatez M.F., Webb A., Crumpton J.C., Webby R.J. (2013). Long-term vaccine-induced heterologous protection against H5N1 influenza viruses in the ferret model. Influenza Other Respir. Viruses.

[45] Middleton D., Rockman S., Pearse M., Barr I., Lowther S., Klippel J., Ryan D., Brown L. (2009). Evaluation of vaccines for H5N1 influenza virus in ferrets reveals the potential for protective single-shot immunization. J. Virol..

[46] Galli G., Hancock K., Hoschler K., DeVos J., Praus M., Bardelli M., Malzone C., Castellino F., Gentile C., McNally T. (2009). Fast rise of broadly cross-reactive antibodies after boosting long-lived human memory B cells primed by an MF59 adjuvanted prepandemic vaccine. Proc. Natl. Acad. Sci. USA.

[47] Goji N.A., Nolan C., Hill H., Wolff M., Noah D.L., Williams T.B., Rowe T., Treanor J.J. (2008). Immune responses of healthy subjects to a single dose of intramuscular inactivated influenza A/Vietnam/1203/2004 (H5N1) vaccine after priming with an antigenic variant. J. Infect. Dis..

[48] Fonville J.M., Wilks S.H., James S.L., Fox A., Ventresca M., Aban M., Xue L., Jones T.C., Le N.M.H., Pham Q.T. (2014). Antibody landscapes after influenza virus infection or vaccination. Science.

[49] Ducatez M.F., Bahl J., Griffin Y., Stigger-Rosser E., Franks J., Barman S., Vijaykrishna D., Webb A., Guan Y., Webster R.G. (2011). Feasibility of reconstructed ancestral H5N1 influenza viruses for cross-clade protective vaccine development. Proc. Natl. Acad. Sci. USA.

[50] Laddy D.J., Yan J., Corbitt N., Kobinger G.P., Weiner D.B. (2007). Immunogenicity of novel consensus-based DNA vaccines against avian influenza. Vaccine.

[51] Laddy D.J., Yan J., Khan A.S., Andersen H., Cohn A., Greenhouse J., Lewis M., Manischewitz J., King L.R., Golding H. (2009). Electroporation of synthetic DNA antigens offers protection in nonhuman primates challenged with highly pathogenic avian influenza virus. J. Virol..

[52] Laddy D.J., Yan J., Kutzler M., Kobasa D., Kobinger G.P., Khan A.S., Greenhouse J., Sardesai N.Y., Draghia-Akli R., Weiner D.B. (2008). Heterosubtypic protection against pathogenic human and avian influenza viruses via in vivo electroporation of synthetic consensus DNA antigens. PLoS ONE.

[53] Giles B.M., Ross T.M. (2011). A computationally optimized broadly reactive antigen (COBRA) based H5N1 VLP vaccine elicits broadly reactive antibodies in mice and ferrets. Vaccine.

[54] Crevar C.J., Carter D.M., Lee K.Y., Ross T.M. (2015). Cocktail of H5N1 COBRA HA vaccines elicit protective antibodies against H5N1 viruses from multiple clades. Hum. Vaccin. Immunother..

[55] Webby R.J., Weaver E.A. (2015). Centralized consensus hemagglutinin genes induce protective immunity against H1, H3 and H5 influenza viruses. PLoS ONE.

[56] Koel B.F., van der Vliet S., Burke D.F., Bestebroer T.M., Bharoto E.E., Yasa I.W., Herliana I., Laksono B.M., Xu K., Skepner E. (2014). Antigenic variation of clade 2.1 H5N1 virus is determined by a few amino acid substitutions immediately adjacent to the receptor binding site. MBio.

[57] De Vries R.P., Smit C.H., de Bruin E., Rigter A., de Vries E., Cornelissen L.A., Eggink D., Chung N.P., Moore J.P., Sanders R.W. (2012). Glycan-dependent immunogenicity of recombinant soluble trimeric hemagglutinin. J. Virol..

[58] Tate M.D., Job E.R., Deng Y.M., Gunalan V., Maurer-Stroh S., Reading P.C. (2014). Playing hide and seek: How glycosylation of the influenza virus hemagglutinin can modulate the immune response to infection. Viruses.

[59] Wang C.C., Chen J.R., Tseng Y.C., Hsu C.H., Hung Y.F., Chen S.W., Chen C.M., Khoo K.H., Cheng T.J., Cheng Y.S. (2009). Glycans on influenza hemagglutinin affect receptor binding and immune response. Proc. Natl. Acad. Sci. USA.

[60] Eggink D., Goff P.H., Palese P. (2014). Guiding the immune response against influenza virus hemagglutinin toward the conserved stalk domain by hyperglycosylation of the globular head domain. J. Virol..

[61] Lin S.C., Liu W.C., Jan J.T., Wu S.C. (2014). Glycan masking of hemagglutinin for adenovirus vector and recombinant protein immunizations elicits broadly neutralizing antibodies against H5N1 avian influenza viruses. PLoS ONE.

[62] Ekiert D.C., Kashyap A.K., Steel J., Rubrum A., Bhabha G., Khayat R., Lee J.H., Dillon M.A., O’Neil R.E., Faynboym A.M. (2012). Cross-neutralization of influenza a viruses mediated by a single antibody loop. Nature.

[63] Hutter J., Rodig J.V., Hoper D., Seeberger P.H., Reichl U., Rapp E., Lepenies B. (2013). Toward animal cell culture-based influenza vaccine design: Viral hemagglutinin N-glycosylation markedly impacts immunogenicity. J. Immunol..

[64] Wang W., Lu B., Zhou H., Suguitan A.L., Cheng X., Subbarao K., Kemble G., Jin H. (2010). Glycosylation at 158N of the hemagglutinin protein and receptor binding specificity synergistically affect the antigenicity and immunogenicity of a live attenuated H5N1 A/Vietnam/1203/2004 vaccine virus in ferrets. J. Virol..

[65] Sun X., Cao W., Pappas C., Liu F., Katz J.M., Tumpey T.M. (2014). Effect of receptor binding specificity on the immunogenicity and protective efficacy of influenza virus A H1 vaccines. Virology.

[66] Hoffmann E., Lipatov A.S., Webby R.J., Govorkova E.A., Webster R.G. (2005). Role of specific hemagglutinin amino acids in the immunogenicity and protection of H5N1 influenza virus vaccines. Proc. Natl. Acad. Sci. USA.

[67] Burke D.F., Smith D.J. (2014). A recommended numbering scheme for influenza A HA subtypes. PLoS ONE.

[68] Krenn B.M., Egorov A., Romanovskaya-Romanko E., Wolschek M., Nakowitsch S., Ruthsatz T., Kiefmann B., Morokutti A., Humer J., Geiler J. (2011). Single HA2 mutation increases the infectivity and immunogenicity of a live attenuated H5N1 intranasal influenza vaccine candidate lacking NS1. PLoS ONE.

[69] Farnsworth A., Cyr T.D., Li C., Wang J., Li X. (2011). Antigenic stability of H1N1 pandemic vaccines correlates with vaccine strain. Vaccine.

[70] Koel B.F., Burke D.F., Bestebroer T.M., van der Vliet S., Zondag G.C., Vervaet G., Skepner E., Lewis N.S., Spronken M.I., Russell C.A. (2013). Substitutions near the receptor binding site determine major antigenic change during influenza virus evolution. Science.

[71] Schotsaert M., De Filette M., Fiers W., Saelens X. (2009). Universal M2 ectodomain-based influenza A vaccines: Preclinical and clinical developments. Expert Rev. Vaccines.

[72] Krammer F., Palese P. (2013). Influenza virus hemagglutinin stalk-based antibodies and vaccines. Curr. Opin. Virol..

[73] Marcelin G., DuBois R., Rubrum A., Russell C.J., McElhaney J.E., Webby R.J. (2011). A contributing role for anti-neuraminidase antibodies on immunity to pandemic H1N1 2009 influenza A virus. PLoS ONE.

[74] Wohlbold T.J., Nachbagauer R., Xu H., Tan G.S., Hirsh A., Brokstad K.A., Cox R.J., Palese P., Krammer F. (2015). Vaccination with adjuvanted recombinant neuraminidase induces broad heterologous, but not heterosubtypic, cross-protection against influenza virus infection in mice. MBio.

[75] El Bakkouri K., Descamps F., De Filette M., Smet A., Festjens E., Birkett A., Van Rooijen N., Verbeek S., Fiers W., Saelens X. (2011). Universal vaccine based on ectodomain of matrix protein 2 of influenza A: Fc receptors and alveolar macrophages mediate protection. J. Immunol..

[76] DiLillo D.J., Tan G.S., Palese P., Ravetch J.V. (2014). Broadly neutralizing hemagglutinin stalk-specific antibodies require FcγR interactions for protection against influenza virus in vivo. Nat. Med..

[77] DiLillo D.J., Palese P., Wilson P.C., Ravetch J.V. (2016). Broadly neutralizing anti-influenza antibodies require Fc receptor engagement for in vivo protection. J. Clin. Invest..

[78] Marcelin G., Sandbulte M.R., Webby R.J. (2012). Contribution of antibody production against neuraminidase to the protection afforded by influenza vaccines. Rev. Med. Virol..

[79] Altenburg A.F., Rimmelzwaan G.F., de Vries R.D. (2015). Virus-specific T cells as correlate of (cross-)protective immunity against influenza. Vaccine.

[80] Hillaire M.L., Vogelzang-van Trierum S.E., Kreijtz J.H., de Mutsert G., Fouchier R.A., Osterhaus A.D., Rimmelzwaan G.F. (2013). Human T-cells directed to seasonal influenza a virus cross-react with 2009 pandemic influenza a (H1N1) and swine-origin triple-reassortant H3N2 influenza viruses. J. Gen. Virol..

[81] Kreijtz J.H., de Mutsert G., van Baalen C.A., Fouchier R.A., Osterhaus A.D., Rimmelzwaan G.F. (2008). Cross-recognition of avian H5N1 influenza virus by human cytotoxic T-lymphocyte populations directed to human influenza a virus. J. Virol..

[82] Van de Sandt C.E., Kreijtz J.H., de Mutsert G., Geelhoed-Mieras M.M., Hillaire M.L., Vogelzang-van Trierum S.E., Osterhaus A.D., Fouchier R.A., Rimmelzwaan G.F. (2014). Human cytotoxic T lymphocytes directed to seasonal influenza A viruses cross-react with the newly emerging H7N9 virus. J. Virol..

[83] Sridhar S., Begom S., Bermingham A., Hoschler K., Adamson W., Carman W., Bean T., Barclay W., Deeks J.J., Lalvani A. (2013). Cellular immune correlates of protection against symptomatic pandemic influenza. Nat. Med..

[84] He X.S., Holmes T.H., Zhang C., Mahmood K., Kemble G.W., Lewis D.B., Dekker C.L., Greenberg H.B., Arvin A.M. (2006). Cellular immune responses in children and adults receiving inactivated or live attenuated influenza vaccines. J. Virol..

[85] Karron R.A., Talaat K., Luke C., Callahan K., Thumar B., Dilorenzo S., McAuliffe J., Schappell E., Suguitan A., Mills K. (2009). Evaluation of two live attenuated cold-adapted H5N1 influenza virus vaccines in healthy adults. Vaccine.

[86] Pitisuttithum P., Boonnak K., Chamnanchanunt S., Puthavathana P., Luvira V., Lerdsamran H., Kaewkungwal J., Lawpoolsri S., Thanachartwet V., Silachamroon U. (2017). Safety and immunogenicity of a live attenuated influenza H5 candidate vaccine strain A/17/turkey/Turkey/05/133 H5N2 and its priming effects for potential pre-pandemic use: A randomised, double-blind, placebo-controlled trial. Lancet Infect. Dis..

[87] Baz M., Boonnak K., Paskel M., Santos C., Powell T., Townsend A., Subbarao K. (2015). Nonreplicating influenza a virus vaccines confer broad protection against lethal challenge. MBio.

[88] Suguitan A.L., McAuliffe J., Mills K.L., Jin H., Duke G., Lu B., Luke C.J., Murphy B., Swayne D.E., Kemble G. (2006). Live, attenuated influenza A H5n1 candidate vaccines provide broad cross-protection in mice and ferrets. PLoS Med..

[89] Talaat K.R., Luke C.J., Khurana S., Manischewitz J., King L.R., McMahon B.A., Karron R.A., Lewis K.D., Qin J., Follmann D.A. (2014). A live attenuated influenza A(H5N1) vaccine induces long-term immunity in the absence of a primary antibody response. J. Infect. Dis..

[90] Peng Y., Wang B., Talaat K., Karron R., Powell T.J., Zeng H., Dong D., Luke C.J., McMichael A., Subbarao K. (2015). Boosted influenza-specific T cell responses after H5N1 pandemic live attenuated influenza virus vaccination. Front. Immunol..

[91] Ren Z., Ji X., Meng L., Wei Y., Wang T., Feng N., Zheng X., Wang H., Li N., Gao X. (2015). H5N1 influenza virus-like particle vaccine protects mice from heterologous virus challenge better than whole inactivated virus. Virus Res..

[92] Smith L.R., Wloch M.K., Ye M., Reyes L.R., Boutsaboualoy S., Dunne C.E., Chaplin J.A., Rusalov D., Rolland A.P., Fisher C.L. (2010). Phase 1 clinical trials of the safety and immunogenicity of adjuvanted plasmid DNA vaccines encoding influenza A virus H5 hemagglutinin. Vaccine.

[93] Stickl H.A. (1974). Smallpox vaccination and its consequences: First experiences with the highly attenuated smallpox vaccine “MVA”. Prev. Med..

[94] Altenburg A.F., Kreijtz J.H., de Vries R.D., Song F., Fux R., Rimmelzwaan G.F., Sutter G., Volz A. (2014). Modified vaccinia virus ankara (MVA) as production platform for vaccines against influenza and other viral respiratory diseases. Viruses.

[95] De Vries R.D., Rimmelzwaan G.F. (2016). Viral vector-based influenza vaccines. Hum. Vaccines Immunother..

[96] Kreijtz J.H., Suezer Y., de Mutsert G., van Amerongen G., Schwantes A., van den Brand J.M., Fouchier R.A., Lower J., Osterhaus A.D., Sutter G. (2009). MVA-based H5N1 vaccine affords cross-clade protection in mice against influenza A/H5N1 viruses at low doses and after single immunization. PLoS ONE.

[97] Kreijtz J.H., Suezer Y., van Amerongen G., de Mutsert G., Schnierle B.S., Wood J.M., Kuiken T., Fouchier R.A., Lower J., Osterhaus A.D. (2007). Recombinant modified vaccinia virus ankara-based vaccine induces protective immunity in mice against infection with influenza virus H5N1. J. Infect. Dis..

[98] Hessel A., Schwendinger M., Holzer G.W., Orlinger K.K., Coulibaly S., Savidis-Dacho H., Zips M.L., Crowe B.A., Kreil T.R., Ehrlich H.J. (2011). Vectors based on modified vaccinia ankara expressing influenza H5N1 hemagglutinin induce substantial cross-clade protective immunity. PLoS ONE.

[99] Kreijtz J.H., Suezer Y., de Mutsert G., van den Brand J.M., van Amerongen G., Schnierle B.S., Kuiken T., Fouchier R.A., Lower J., Osterhaus A.D. (2009). Recombinant modified vaccinia virus Ankara expressing the hemagglutinin gene confers protection against homologous and heterologous H5N1 influenza virus infections in macaques. J. Infect. Dis..

[100] Xiao H., Liu L., Zhu Q., Tan Z., Yu W., Tang X., Zhan D., Du Y., Wang H., Liu D. (2013). A replicating modified vaccinia tiantan strain expressing an avian-derived influenza H5N1 hemagglutinin induce broadly neutralizing antibodies and cross-clade protective immunity in mice. PLoS ONE.

[101] Kreijtz J.H., Goeijenbier M., Moesker F.M., van den Dries L., Goeijenbier S., De Gruyter H.L., Lehmann M.H., Mutsert G., van de Vijver D.A., Volz A. (2014). Safety and immunogenicity of a modified-vaccinia-virus-Ankara-based influenza A H5n1 vaccine: A randomised, double-blind phase 1/2a clinical trial. Lancet Infect. Dis..

[102] De Vries R.D., De Gruyter H.L., Bestebroer T.M., Pronk M., Fouchier R.A., Osterhaus A.D., Sutter G., Kreijtz J.H., Rimmelzwaan G.F. (2015). Induction of influenza (H5N8) antibodies by modified vaccinia virus ankara H5N1 vaccine. Emerg. Infect. Dis..

[103] Kreijtz J.H., Wiersma L.C., De Gruyter H.L., Vogelzang-van Trierum S.E., van Amerongen G., Stittelaar K.J., Fouchier R.A., Osterhaus A.D., Sutter G., Rimmelzwaan G.F. (2015). A single immunization with modified vaccinia virus ankara-based influenza virus H7 vaccine affords protection in the influenza A(H7N9) pneumonia ferret model. J. Infect. Dis..

[104] Florek N.W., Weinfurter J.T., Jegaskanda S., Brewoo J.N., Powell T.D., Young G.R., Das S.C., Hatta M., Broman K.W., Hungnes O. (2014). Modified vaccinia virus ankara encoding influenza virus hemagglutinin induces heterosubtypic immunity in macaques. J. Virol..

[105] Poon L.L., Leung Y.H., Nicholls J.M., Perera P.Y., Lichy J.H., Yamamoto M., Waldmann T.A., Peiris J.S., Perera L.P. (2009). Vaccinia virus-based multivalent H5N1 avian influenza vaccines adjuvanted with IL-15 confer sterile cross-clade protection in mice. J. Immunol..

[106] Kamlangdee A., Kingstad-Bakke B., Anderson T.K., Goldberg T.L., Osorio J.E. (2014). Broad protection against avian influenza virus by using a modified vaccinia ankara virus expressing a mosaic hemagglutinin gene. J. Virol..

[107] Potter C.W., Oxford J.S. (1979). Determinants of immunity to influenza infection in man. Br. Med. Bull..

[108] Luke C.J., Subbarao K. (2014). Improving pandemic H5N1 influenza vaccines by combining different vaccine platforms. Expert Rev. Vaccines.

